# Bone metastases in hepatoblastoma, an unusual presentation. Case report and review of the literature

**DOI:** 10.1016/j.radcr.2022.08.025

**Published:** 2022-09-13

**Authors:** Viviana Barragan, Maria Camila Escudero, Ivette C. Jimenez, Catalina Correa, Juan Pablo Luengas

**Affiliations:** a Universidad Militar Nueva Granada, Hospital Militar Central, Bogotá, DC, Colombia; b Universidad de La Sabana, Bogotá, DC, Colombia; c Universidad El Bosque, Bogotá, DC, Colombia; d Hospital Militar Central, Bogotá D.C, Colombia; e Instituto Nacional de Cancerología, Bogotá́, DC, Colombia

**Keywords:** Hepatoblastoma, Neoplasm staging, Alpha-fetoprotein, Neoplasm metastasis, Bone, Therapeutics

## Abstract

Primary liver tumors are rare in childhood. Hepatoblastoma is the most prevalent and has a variable clinical presentation. The initial approach requires clinical suspicion, histopathological confirmation, and measurement of AFP levels, in addition to PRETEXT staging by abdominal computed tomography. PET-CT is useful in metastatic disease for diagnosis and evaluation of therapeutic response. Pulmonary metastases at the time of diagnosis are frequent, while bone metastases are rare. We present the case of an infant with a history of metastatic hepatoblastoma, multiple relapses, and poor response to multimodal management. The patient had bone metastases demonstrated by PET-CT imaging.

## Introduction

Primary liver tumors are infrequent in childhood. However, Hepatoblastoma (HB) is the most common malignant tumor in children under 3 years of age, representing 1%-2% of all malignant tumors in this population group, with an incidence of 1.5 to 5 cases per 1,000,000 children [1]. Although HB etiology is unknown, its association with familial adenomatous polyposis, Beckwith-Wiedemann syndrome, and very low birth weight (less than 1000 grams) is documented [2].

Classically, HB presents with abdominal mass, which can be associated with pain, distension, anorexia, and in rarer cases, acute abdomen (tumor rupture) and anemia [3,4]. In most cases, finding a palpable abdominal mass, a liver-dependent tumor on imaging, and serum elevation of alpha-fetoprotein (AFP) is diagnostic of HB. Confirmation by histopathological study of a biopsy or primary tumor resection is necessary [5].

The histological tumor type, AFP level, the presence of metastasis at the time of diagnosis, response to chemotherapy, and the PRETEXT and POST TEXT classification of the tumor significantly impact the prognosis and type of the disease. In addition, such variables allow standardization of treatment and follow-up [3,5].

In recent decades, survival of HB has increased to 70%-80%. However, the prognosis is less favorable in high-risk pediatric patients with unresectable tumors at diagnosis, extrahepatic involvement, or distant metastases [5,6]. Furthermore, around 20% of patients have pulmonary metastases at diagnosis, and recurrence of HB is usually pulmonary.

There is little literature on metastatic involvement caused by HB, such as bone, adrenal gland, or central nervous system (CNS) metastases [5,7]. Therefore, we present the case of an infant with unresectable PRETEXT III stage hepatoblastoma at diagnosis, recurrent on 2 occasions, and with bone metastases as an unusual presentation of HB.

## Materials and methods

An 18-month-old male patient consults for a 3-month clinical history of progressive abdominal distension and occasional vomit associated with respiratory distress. Physical examination revealed an enlarged liver and pain on abdominal palpation. There were no peritoneal signs. A computed tomography (CT) of the abdomen showed a liver-dependent tumor of heterogeneous appearance, with partially defined contours and hypodense images at the center of approximately 11.7 mm × 9.7 mm × 11.9 mm, occupying a large part of the right hepatic lobe, with multifocal involvement. AFP values greater than 500. Pulmonary metastasis was evident in the staging images. The patient underwent liver biopsy with histopathological confirmation of mixed Hepatoblastoma, epithelial and mesenchymal, receiving PRETEXT III staging. Surgical resection was not considered, so the patient received 5 cycles of neoadjuvant chemotherapy with Vincristine, Cisplatin, and Fluorouracil. Following chemotherapy, the images showed a 60% decrease in tumor size, with POST TEXT staging II and the control of pulmonary metastases. Right hepatectomy was performed with good postoperative outcomes, and AFP levels declined.

One year after local control of the disease, a new elevation of AFP was documented, with the finding of pulmonary nodules by thoracoabdominal CT, thus evidencing the first relapse. Bilateral sequential pulmonary metastasectomy was performed. Sixteen months later, he presented a second relapse in the right lung. The patient underwent a new metastasectomy, resulting in disease control and the levels of AFP decreased to 1.26.

**Fig. 1:**
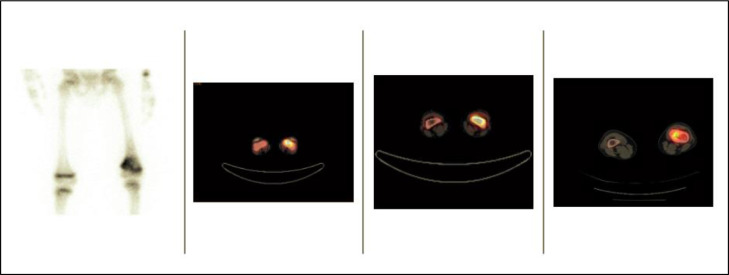
PET-CT: Hypermetabolic lesion located in the distal diaphysis of the left femur with an intramedullary location, with slight loss in a portion of the cortical bone in its anterior aspect, suggestive of tumor involvement (arrow).

The following year, the progressive elevation of AFP up to 259 was documented. Imaging did not show thoracoabdominal lesions. However, positron emission tomography (PET-CT) was performed due to pain in the left lower limb. A hypermetabolic lesion in the left distal femur was detected, and a bone biopsy confirmed metastatic involvement by epithelioid tumor with an immune profile that favored hepatoblastoma. Thus, the patient had a third relapse with bone metastasis (Fig. 1).

Based on the failure of the third-line chemotherapeutic treatment, low probability of cure, and insufficient benefit of limb salvage surgery in this case, a multidisciplinary team ruled out the possibility of curative treatment. Instead, palliative radiotherapy in the distal femur was indicated. The patient received external radiotherapy with the energy of 6 MV in 3 GY fractionation up to a total dose of 30 GY in the lesion area and margins. She attended a bi-monthly follow-up until her death, which occurred due to the progression of the disease 11 months after the bone relapse.

## Discussion

Hepatoblastoma is the most prevalent hepatic tumor in the pediatric age, representing 70% of malignant liver tumors. Hepatocellular carcinoma and hepatic embryonal sarcomas comprise less than 30% [1,3]. Most of them are sporadic and occur in patients with no pathological history. HB is associated with genetic syndromes such as Beckwith-Wiedemann, Sotos, Simpson-Golabi-Behmel, and familial adenomatous polyposis (FAP) [1].

The overall incidence of HB is less than 5 cases per million, with recent reports estimating an annual increase of 2.7%, related to the increased survival of very low birth weight infants and the implementation of chemotherapy regimens with better clinical outcomes [4,5]. Its clinical presentation is variable and nonspecific. Ninety percent of cases occur before 5 years of age [3].

International pediatric liver tumor groups such as SIOPEL, CHIC, and COG suggest an initial approach to patients with suspected HB consisting of a 3-stage CT scan of the abdomen or MRI with hepatospecific contrast to obtain a PRETEXT staging [7,8]. This classification system defines 4 stages concerning the anatomical affectation of the liver and the involvement of other structures.

AFP is the tumor marker for HB, both diagnosis and follow-up: levels above 100 ng/mL at diagnosis are associated with 5-year survival rates below 18%. Imaging and age are also fundamental in classifying patients by risk groups and estimating their long-term survival. In addition, a biopsy is helpful for histological confirmation of the tumor [3,9].

Complete tumor resection is the treatment of choice for patients with early-stage or low-risk diseases susceptible to surgical management. Such management has good results in terms of prognosis and recovery rates, and the 5-year survival is up to 100%. Meanwhile, tumors in more advanced or unresectable stages, such as PRETEXT III and IV, have a 5-year survival of 78 and 44%, respectively [1,^10^,11].

The presence of metastases at diagnosis is frequent and is an independent predictor of poor prognosis associated with lower overall survival (up to 25%). However, metastatic disease is not an absolute contraindication for surgical treatment or therapeutic liver transplantation. Therefore, evaluating the presence of poor prognostic factors, specifically unresolved pulmonary metastases, is essential to guide this therapeutic possibility [3,^6^,9].

The lung is the most frequent site of distant metastasis, followed by the brain, bone, and other less frequent sites [12]. For example, Zhi et al. found that 132 of 316 children with HB had distant metastases, mainly to the lung in 80%, followed by 6% with intracranial metastases, 4.5% bone, 3% diaphragmatic, 3% to the right atrium, 3% to the pleura, 1.5% intraspinal, 1.5% to the kidney and adrenal gland, and 0.75% had intestinal metastases [3]. Likewise, Zhang et al describe, in a retrospective study, that 48% of the patients had distant metastases at diagnosis: 75% were pulmonary, 20% vascular, 34% intrahepatic, and 12% bone metastases [5,13].

Different therapeutic options are available depending on tumor stage and metastatic state, ranging from chemotherapy (neo/adjuvant), chemoembolization, tumor resection, metastasectomies, radiofrequency ablation, and liver transplant [5,^10^,14]. In patients with incomplete tumor resection or with recurrent disease, re-staging is required to determine the need for new tumor resection or liver transplantation [15]. Matsunaga et al reported that 20 children with distant metastases who underwent a total lumpectomy had a 2-year survival rate of 80%, while those who did not undergo complete resection died [11].

In contrast, neoadjuvant chemotherapy in recent years has improved overall survival and response rates to treatment. For example, in a study, Angelico et al described patients with HB and pulmonary metastases who underwent neoadjuvant chemotherapy: 43% of the lesions disappeared, and only 27% of the cases required metastasectomy [9].

The indications for surgical management of pulmonary metastases vary according to the resectability of the primary tumor [9]. Surgery is indicated to remove the affected hepatic segments of patients with a good response to chemotherapy. Notwithstanding, in patients with no response to chemotherapy, pulmonary metastasectomy can be performed, and it offers a long-term survival of 88% [10,11].

Additionally, Zsiros et al. reported excellent results with the SIOPEL-4 protocol (intensive preoperative chemotherapy and radical surgery for high-risk HB), in which the inclusion of cisplatin in neoadjuvant chemotherapy increased the response to 97%, decreasing the requirement for metastasectomies [16]. In contrast, children with a metastatic disease whose primary tumor is unresectable or PRETEXT stage IV, who, after receiving neoadjuvant chemotherapy, are candidates for metastasectomy or liver transplantation, have had similar results with high 5-year survival rates [9,16].

Within the therapeutic arsenal, liver transplantation has demonstrated high survival rates (up to 85%) in patients with locally advanced HB, incomplete tumor resection, or insufficient liver remnant associated with acute liver failure. However, liver transplant has serious complications that can affect patients' quality of life [6]. In contrast, there are few reports of HB with bone metastases, so there is no consensus on the management and survival of these patients.

Diagnostic imaging in HB has evolved. Until some years ago, bone scintigraphy with technetium 99m was part of the study in patients with HB. However, most reports showed abnormalities in bone activity, especially concerning the loss of differentiation metaphysis—physis, secondary to osteopenia, without clearly defining bone metastases. Therefore, bone scintigraphy is no longer part of the necessary studies in asymptomatic patients [8,17]. Currently, CT is the imaging of choice in preoperative diagnosis and staging. PET-CT with fluorodeoxyglucose (F-FDG) optimizes anatomical localization of the lesions and response to treatment, especially in the early detection of tumor recurrence or metastasis. PET-CT has a sensitivity of 98% and specificity of 56% in bone metastasis [18], [19], [20] and is especially useful in HB as it provides an accurate image of the tumor's metabolic activity: HB has a high content of cellular glycogen with a great glycolytic activity [18], [19], [20], [21].

## Conclusions

Hepatoblastoma is the most common malignant liver tumor in children under 3 years of age. Bone metastases are rare, and few cases with metachronous metastases to both lung and bone are available in the literature. Therefore, we present a case report of a patient with 2 pulmonary relapses and the third one with bone involvement. The lack of extension studies on bone may be related to a low diagnostic rate. We consider that using PET CT in re-staging may be helpful since it has demonstrated good sensitivity and specificity rates for the timely diagnosis of these lesions. Further studies are needed involving patients with HB and bone metastases to reach a consensus on the best treatment approach for these patients.

## Patient consent

Authors inform that obtained voluntarily written consent from the patient's mother (legal representative) to publish this case report. She declared to sign the consent with full understanding of all implications for the patient. We declare that there is not identifiable information or images for our patient.
